# Sexual health interventions with social marketing approach targeting young people: a scoping review

**DOI:** 10.1093/heapro/daae106

**Published:** 2024-08-16

**Authors:** Hanna Putkonen, Hanna Kallio, Jari Kylmä, Tiina Rissanen, Marjorita Sormunen

**Affiliations:** Faculty of Health Sciences, Department of Public Health and Clinical Nutrition, University of Eastern Finland, Yliopistonranta 8, Kuopio, 70210, Finland; Faculty of Medicine, Department of Nursing Science, University of Turku, Medisiina B, Kiinamyllynkatu 10, Turku, 20520,Finland; Faculty of Social Sciences, Unit of Health Sciences, Nursing Science, University of Tampere, Kalevantie 5, Tampere, 33100, Finland; Faculty of Health Sciences, Department of Public Health and Clinical Nutrition, University of Eastern Finland, Yliopistonranta 8, Kuopio, 70210, Finland; Faculty of Health Sciences, Department of Public Health and Clinical Nutrition, University of Eastern Finland, Yliopistonranta 8, Kuopio, 70210, Finland

**Keywords:** young people, health education, health promotion, sexual health, social marketing

## Abstract

In a fast-paced digital and global environment, sexual education must keep up with young people’s sexual health needs. Social marketing is an approach that has been used in sexual health promotion for young people. The objective of the scoping review is to identify and map the use of social marketing in sexual health promotion for young people. Specifically, the content, delivery methods and effects of interventions on sexual health were researched. Six databases were systemically searched to capture the relevant peer-reviewed quantitative, qualitative and mixed methods articles without time restrictions that provided evidence of sexual health-related social marketing interventions targeting young people aged 11–25. An inductive and deductive content analysis was performed. Nineteen studies were included in the data. The content of interventions was dominated by sexual risks and risk prevention, focusing particularly on sexually transmitted diseases, unwanted pregnancies and sexual violence. Additionally, interventions included topics of morals of sexual relationships and changes in the body. The delivery of interventions occurred through various media channels, events and activities, while the effects of interventions were monitored as improvements in sexual perceptions and sexual behaviour, limited gender-related effects, limited evidence of intervention attributed to behaviour and effects in different age groups. The social marketing approach was mostly preventive and concentrated on the risks, whereas the delivery methods were diverse and creative, combining modern and already well-established channels. Sexuality should be seen comprehensively, and interventions should respond to the full range of young people’s needs.

**Contribution to Health Promotion**

- This review indicates that social marketing interventions on young people’s sexual health emphasize risks. Other essential sex education themes such as social and mental well-being, equality and respect and love and relationships could be addressed more profoundly.
- The existing studies suggest that social marketing offers a valid approach to sexual health promotion with versatile, innovative and multi-channelled implementation techniques.
- The effects of sexual health social marketing interventions targeting young people can be seen in behaviour and perceptions and are age-related. Gender associations on effects should be studied further.

## BACKGROUND

Amidst significant physiological, social and psychological changes, and during the crucial development of sexuality and sexual behaviours, adolescents begin to form various bonds, including dating relationships and friendships (Wildsmith and Vaughn, 2013; Kar *et al*., 2015; World Health Organization, 2021). Sexuality is defined as a fundamental aspect of being human, and it encompasses gender identities and roles, sex, sexual orientation, eroticism, pleasure and reproduction. Sexual health, in turn, covers a state of physical, emotional, mental and social well-being about sexuality (World Health Organization, 2021). The sexual development of young people is influenced by biological and psychological factors, but also other elements such as legal, political, ethical, philosophical, spiritual and moral values, and media (Harris, 2011; Merrick *et al*., 2013).

The state of adolescents’ sexual health varies greatly across and within countries (Liang *et al*., 2019). Within the last three decades, significant progress has been made with a decline across sexual health indicators such as adolescent pregnancy (Loaiza and Liang, 2013), child marriage (United Nations Children’s Fund, 2018) and female genital mutilation (Shell-Duncan and Naik, 2016). However, sexual health issues including inequalities in key indicators of adolescent health, increase in intimate partner violence, reproductive cancer and sexually transmitted infections (STIs) remain a great public health concern (Forsyth and Rogstad, 2015; Liang *et al*., 2019). Young people are particularly vulnerable to face challenges that disadvantage their sexual health (Slater and Robinson, 2014). In the future, challenges in ensuring access to reproductive health care and education and addressing embedded gender norms will continue to exist. Pandemics, conflicts and climate change are adding to the severity, frequency and impact of disruptions (Mehta and Seeley, 2020).

Sexual education has an important role in tackling the sexual health challenges of young people (Council of Europe, 2020). Through sexual education provided by families, schools, health care and various community actors, young people can access and gain the information, tools and skills that help them better adapt to biological and psychological changes (Council of Europe, 2020; Pakarinen *et al*., 2020; World Health Organization, 2021). Via sexual education, one’s sexual health literacy skills—the ability to understand sexual health information and application of that information, decreasing the risk of (STIs) and providing various benefits beyond—can be strengthened (World Health Organization, 2016). There is an increasing understanding that young people are ‘knowledgeable actors’ in the field of sexual education and that it should be practised *with* rather than *for* them (Coll *et al*., 2018). Utilizing comprehensive sexual education, young people are capable of making safe and responsible choices while enjoying satisfying relationships (World Health Organization, 2021).

Social marketing, a systematic approach in which the components of commercial marketing are integrated into public health strategies (Lee *et al*., 2023), is one approach that has been used in sexual health promotion for young people. This approach aims to change or maintain people’s behaviour, which benefits individuals and society (National Social Marketing Centre, 2023). Social marketing is based on research and a comprehensive strategy, and it is not just a communication campaign, even if communication or messaging is often the most visible part of social marketing for the audience (Weinreich, 2011).

In aiming for social good, social marketing uses marketing mix strategies, and depending on the views, the marketing mix consists of the four Ps (Kotler and Zaltman, 1971) or ‘more Ps’ (Weinreich, 2011). The four Ps refer to product, price, place and promotion and come from the traditional marketing practice. Product designates to the benefits of performing the desired behaviour, whereas price points out the cost of adopting the behaviour. Place refers to convenient access for the audience to engage in the targeted behaviours, while promotion is about persuasive communication highlighting benefits (Weinreich, 2011). Communication channels for promotional messages can be classified as Internet, broadcast media, print media and out-of-home media (BBA Mantra, 2017).

‘More Ps’, the addition of social marketing to the marketing mix, indicate publics, partnership, policy and purse strings. In social marketing, various audiences are involved in interventions with different roles, and publics refer to these external groups such as the target audience, and internal groups such as staff and organizations. Partnership and cooperation with other groups in the community are crucial when considering complex health issues. To support individual behaviour change, the policy level must be treated and influenced by social marketing acts. Finally, purse strings can be seen as one dimension of strategy development as many social marketing programs operate through funds from foundations, governmental grants or donations (Weinreich, 2011).

Social marketing shares some principles with other behaviour change approaches, such as audience orientation, segmentation, behaviour focus and evaluation. However, social marketing has unique principles that distinguish this approach from other forms of behaviour change, such as value exchange, recognition of competition, sustainability and the four Ps of marketing (Lee *et al*., 2023). The type of social marketing interventions is often listed as educational, supportive, design and controlling, and these domains form ‘the intervention mix’. A range of approaches is required as single, isolated interventions can rarely influence behaviour remarkably (National Social Marketing Centre, 2023).

In this audience-centred approach, the actions are strategically designed based on the audience’s needs, values, motivations and concerns (Lee *et al*., 2023). In young people’s sexual health, social marketing strategies have been used to address their sexual health-related misperceptions (Messer *et al*., 2011), promoting STI testing (Wilkinson *et al*., 2016; Riddel et. al 2024), popularizing condom use (Purdy, 2011; Sweat *et al*., 2012) and addressing unintended teenage pregnancies (Wakhisi *et al*., 2011; Ponsford *et al*., 2022).

Although literature reviews about young people’s sexual health promotion in the digital era have been conducted (Sanz-Lorente *et al*., 2018; Martin *et al*., 2020; Engel, 2023), to our knowledge, reviews analysing specifically the content and delivery methods of sexual health social marketing programs targeting young people have not been performed. In response to this, the objective of this scoping review is to identify and map the existing circumstances on how social marketing has been used in sexual health promotion targeting young people aged 11–25 globally. Three questions guided the review: (i) What kind of content is included in social marketing interventions promoting young people’s sexual health; (ii) What kinds of delivery methods are used in social marketing interventions promoting young people’s sexual health and (iii) What kinds of effects do social marketing interventions have on young people’s sexual health?

## METHODS

### Search strategy and selection criteria

For synthesizing evidence, a scoping review (The Joanna Briggs Institute, 2023) was conducted with a systematic literature search of sexual health promotion and social marketing. This scoping review is reported based on the Preferred Reporting Items for Systematic Reviews and Meta-Analyses–Extension for Scoping Reviews (PRISMA-ScR) (Tricco *et al*., 2018). Scoping reviews can be undertaken when the research phenomenon is complex or has not been reviewed comprehensively before. Furthermore, scoping reviews seek to provide in-depth coverage of the available literature and map the key concepts underpinning a research area (Arksey and O’Malley, 2005). As the objective of this study was to identify and map the available evidence, a scoping review was a suitable method (Munn *et al*., 2018). A comprehensive electronic search was conducted from PubMed, Eric, Cinahl, PsycINFO, Scopus and Web of Science. To acquire a comprehensive understanding of the topic that is not much studied, time restrictions for the published articles were not established. The inclusion criteria are presented in Table 1. See Supplementary File S1 for the search strategy.

**Table 1:** The inclusion criteria of the scoping review

	Inclusion criteria
Population	Adolescents and young adults 11–25 years
Geographic area	Unlimited
Intervention	Adolescent sexual health promotion using social marketing approach
Content of articles	Empirical studies The design, implementation, outcomes, effects and/or evaluation of interventions Studies that self-identified themselves as social marketing programs by authors of the original articles
Year of publication	Unlimited
Language	English
Methodology	Qualitative, quantitative and mixed methods
Context	Programs and interventions designed for and implemented in any place and setting
Type	Original peer-reviewed research articles

Quantitative (*n* = 12); qualitative (*n* = 4) and mixed methods (*n* = 3) studies were included with various designs to consider different aspects of the topic.

The literature filling the criteria published by February 2022 was reviewed. Regarding the inclusion criteria, exceptions were made with three included studies (Wagman, 1993; Wilkins and Mak, 2007; Janssen *et al*., 2021) as they targeted young people with a wider age range, up to 29 and 30 years. As the average age fell within the age range of this study, 11–25 years, these three studies were included.

**Fig. 1:**
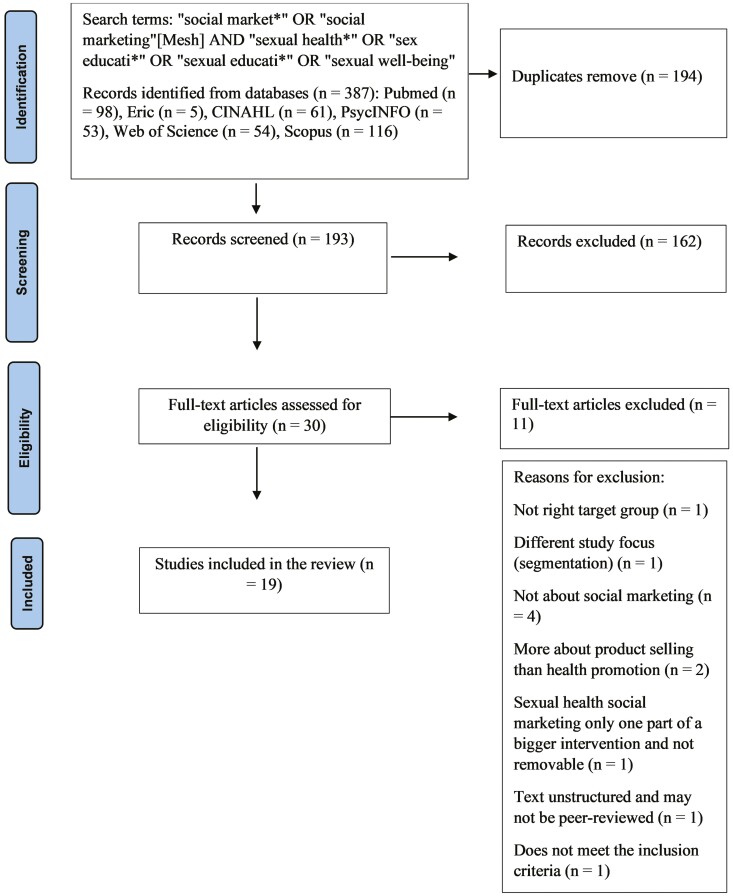
The PRISMA flow diagram of the selection process.

A total of 387 studies were retrieved and imported to a reference management software, RefWorks (Proquest, 2024). After the removal of duplicates, 193 articles were screened. The title, abstract, full-text screening and selection were carried out by two authors (H.P. and H.K.) independently. First, the articles were screened based on their title and abstracts independently. After this screening phase, the article selection was discussed, and all authors finalized the inclusion criteria. When in consensus, the screening continued to the full texts and the practices followed the previous screening phase. Figure 1 depicts the selection process to include articles as part of the scoping review.

### Data analysis

Data analysis was conducted by one author (H.P.) using an inductive and deductive content analysis separately (Kyngäs *et al*., 2019), and the analysis was discussed and finalized in the research group. An inductive content analysis was performed to understand the content and the effectiveness of the interventions (research questions one and three) and a deductive analysis to acknowledge the methods used to deliver the interventions (research question 2). This division was performed due to the lack of or fragmented previous theoretical knowledge for the first-mentioned questions and an available theoretical structure and the possibility of knowledge accumulation for the latter. To get an overview of the selected articles, they were summarized in a data extraction matrix. The interventions were organized on a separate matrix for a more specific review.

All expressions that answered the research questions were extracted. The next phase of the process was organizing the data. This process included open coding, creating categories and abstraction. The open codes were analysed to form generic categories that were further grouped into main categories. Each category was named using content characteristic words (Kyngäs *et al*., 2019).

Inductive content analysis involves collecting and analysing data without preconceived theories, whereas deductive content analysis can be applied when the starting point of the research is earlier theoretical knowledge (Kyngäs *et al*., 2019). In this research, the social marketing principles of the four Ps (Lee *et al*., 2023) and a classification of four media types (BBA Mantra, 2017) were exploited as an analysis foundation for research question 2. Derived from the existing theory, the categories and subcategories were formed, and the codes were grouped according to their similarities and differences. The links between subcategories and main categories were established and the conclusions were drawn from the coded data.

## RESULTS

In total, 19 studies were included in the review (Table 2). Studies were conducted between the years 1993 and 2021, most commonly between 2011 and 2021 (*n* = 12). Studies were performed in eight countries in four continents (North America: *n* = 13, Africa: *n* = 3, Australia: *n* = 2, Asia: *n* = 1). One study was conducted in four countries in Africa (Table 2). The interventions had a strong educational emphasis.

**Table 2:** The included studies of the scoping review

Author, year and country	Study aim	Target population	Methodology and study design	Years of data collection and sample size	Intervention name and components	Outcomes
Agha, 2002 Cameroon, South Africa, Botswana, Guinea	To evaluate the impact of adolescent sexual health social marketing interventions	Adolescents	Quantitative Quasi-experimental design	1994–98 Cameroon baseline (*n* = 1,606) follow-up (*n* = 1,633) South Africa baseline (*n* = 221) follow-up (*n* = 204) Botswana baseline (*n* = 1,002) follow-up (*n* = 2,396) Guinea baseline (*n* = 2,016) follow-up (*n* = 2,005)	Four interventions without name details. Peer education, youth clubs, mass media advertising and the distribution of educational materials. Weekly talk shows on radio	Perceptions, beliefs, behaviour
Andrade *et al*. 2018 USA	Develop and pilot a social marketing intervention for ‘hard-to-reach’ immigrants targeting risk factors for co-occurring youth substance abuse, sexual risk and violence	Latinos aged 12–19 and program staff	Qualitative Four-phase formative research process, campaign development based on findings from one group interview and four focus groups with youth	Years of data collection was not provided. Staff (*n* = 8) Youth (*n* = 35)	Adelante Printed adds, multiplatform social media promotion, contests, expert panel, youth-generated videos, blog posts, text messages, sports and recreation	Substance abuse, sexual risks, violence
Aronowitz *et al*. 2018 USA	To present methods in engaging students in a multi-level intervention aimed at preventing substance abuse, HIV and STIs	College students	Mixed methods Focus groups, post-event surveys, annual evaluations	2015–16 8 focus groups having 3–6 students in each group Post-event surveys (*n* = 208)	No name provided Website, free HIV testing events on campus, wheel of sex game on the campus, lunchtime ‘Health talks’, and annual Sex in the Dark Q&A event	Substance abuse, HIV, STIs
Bull *et al*. 2008 USA	To evaluate effects of a social marketing campaign on awareness of, attitudes towards and use of female as well as male condoms	15- to 25-year-old women	Quantitative Cross-sectional survey Pre- and post-campaign surveys	2004–06 Pre-campaign (*n* = 3,407) Post-campaign (*n* = 3,003)	POWER Print media, posters and takeaway information cards	Awareness of and attitudes towards condoms
Cho *et al*. 2004 USA	To report the use of formative research to develop audience-centred and culturally sensitive messages and report how mixed-media was utilized to reach at-risk populations	Hispanic young adults aged 18–24	Qualitative Focus groups	2001 Family planning service providers (*n* = 35) Young adults (*n* = 22)	The Healthy Talk Multi-communication channels	Unintended pregnancies, STIs
Chou *et al*. 2020 Taiwan	To determine the effectiveness of the intervention to promote adolescent sexual health in junior high schools	Students aged 13–14	Quantitative A one-group pre-test–post-test design	2016–17 (*n* = 1,407)	Starting from love—Go! Go! Go! Representatives from each school, teacher training, formal sex education sessions, consultation mailbox, competitions, debates, learning feedback, role plays, computer animations, quizzes	Sexual knowledge, attitudes
Eastman-Mueller *et al.*, 2019 USA	To examine the association of Get Yourself Tested (GYT), a sexual health social marketing campaign, with several sexual health behaviours	High school and college students	Quantitative Survey	2013 (*n* = 2,329)	Get yourself tested GYT Website, television, print and social media in combination with testing events and concert series	STI testing, communication with healthcare providers
Evans *et al*. 2019 USA	To evaluate campaign exposure and changes in positive youth development (PYD) outcomes	Latinos aged 12–17	Quantitative Three cross-sectional surveys Intervention–comparison groups	2014–16 (*n* = 1,549)	Adelante Outdoor advertising, videos and social media	Substance use, sexual risk taking, violence-related knowledge, attitudes, intentions, risk behaviour
Friedman *et al*. 2014 USA	To present the strategies and outcomes of local GYT campaigns, highlighting the diversity in which a national sexual health campaign is implemented at the local level and identifying challenges and successes	15- to 25-year-old women and partners	Quantitative Local campaign reach and engagement were assessed through the tracking of events, material distribution, media coverage, web and social media metrics and audience participation and event attendance. All sites submitted final reports after the grant period. Each site’s final report included chlamydia testing and positivity data collected during campaign implementation periods and comparable baseline data from the same time in the previous year.	2010–11 Campaigns (*n* = 9)	Get yourself tested GYT Combination of traditional and new media, on-the-ground activities, promotional products and events, social media, websites	Chlamydia screening, treatment
Garbers *et al*. 2016 USA	The study included formative and materials-testing, adaptation of existing campaign materials to be more inclusive of Black and Latino sexual-minority youth, conduct a campaign to promote STD testing at events and through mobile testing and online and social media platforms, evaluation of outreach activities and an outcome evaluation of testing	Black and Latino sexual-minority youth	Qualitative Focus groups Quantitative for outcome evaluation Pre-experimental design	Years of data collection was not provided. (*n* = 299)	Get yourself tested GYT Events, mobile testing and online and social media platforms	STI testing
Habel *et al*. 2015 USA	To investigate the associations between STI testing and the campaign exposure	College students	Quantitative The measures consisted of student completion of a self-report questionnaire, school-level variables and STI testing numbers/positivity. Chi-square and binary regression analyses tested for associations with GYT campaign awareness, STI testing behaviours and STI test results.	2011 (*n* = 1,386)	Get yourself tested GYT Television, print, web and social media in combination with on-the-ground outreach efforts such as testing events and concert series, testing events	STI testing
Janssen *et al*. 2021 Australia	To investigate whether and how the tailored messages reached the intended audience and describe the intervention	Youth aged 15–29	Quantitative	2017–20 (*n* = 1,776)	Down to test DTT Outdoor music festival activations, digital media communications, peer educators, activations in VIP area by trained peers	STI prevention
Meekers 2000 South Africa	To assess the effect of a social marketing program on reproductive health beliefs and behaviour	17- to 20-year-old women	Quantitative Quasi-experimental control group design Pre- and post-intervention surveys	1996–97 (*n* = 430)	The Soweto adolescent reproductive health program Mass media campaign, peer education, targeted condom distribution, radio, TV, posters, T-shirts, buttons	Reproductive health beliefs, behaviour
Van Rossem and Meekers, 2000 Cameroon	Examines the effectiveness of a social marketing program for improving adolescent reproductive health	Youth aged 12–22	Quantitative Quasi-experimental design	1996–97 Baseline (*n* = 1,606) Follow-up (*n* = 1,633)	Horizon Jeunes Youth clubs, mass media promotion, peer education, radio spots, talk shows, meetings, events, condom use demonstration, sketches	Preventive behaviour
Wagman 1993 Canada	Describes the designing and implementing of a public education intervention	Young adults 19–30 years old, emphasis on women	Mixed methods Qualitative and quantitative tools for the baseline knowledge, a randomized pre-test post-test telephone survey, a series of in-depth interviews with bar patrons. Later four focus groups for conceiving the third campaign	This information was not provided.	Condomania Multimedia, advertising, contests, trained volunteers to distribute condoms at nightclubs, follow-up phone, TV, newspapers	Condom use
Wilkins and Mak, 2007 Australia	Evaluation of the Western Australian Department of Health chlamydia campaign	15- to 24-year-olds	Mixed methods Focus test Surveys GP room audit Calculation of website traffic and emails to campaign website	2005 Focus test (*n* = 29) survey (*n* = 122) GP general practice waiting room audit *n* = 43	The chlamydia campaign SMS, website, posters in pubs and clubs and university	Awareness and opinions of a chlamydia campaign
Willoughby 2013 USA	Describes the development of a social marketing campaign promoting a state-based sexual health text message service that allows teens to text a sexual health question directly to a trained health educator	14–18 years olds	Qualitative In-depth interviews and focus groups	Years of data collection was not provided. (*n* = 35)	BrdsNBz Text message service and promotional items: pens, wallet cards with service info	Sexual health SMS service
Willoughby 2015 USA	To assess the effectiveness of an in-school social marketing campaign promoting a sexual health text message service that connects teens directly with a health educator.	14–18 years olds	Mixed methods Explanatory sequential mixed methods design Focus groups and in-depth interviews Three quantitative methods (service use data, a text message-based questionnaire and an in-school online survey)	2012–13 Quantitative (*n* = 2,125) Qualitative (*n* = 18)	BrdsNBz Text message service and promotional items: pens, wallet cards with service info	Sexual health SMS service
Zhang *et al*. 2017 Canada	Describe the implementation of the digital gaming campaign in four locations in movie theatres and report process evaluation indicators for the campaign.	Young adults aged 20–29	Quantitative Web analytics	2014–15 Views (*n* = 548,410) Plays (*n* = 77,149)	Testing is healthy! Mobile application	Use of a Clinic Finder page

Quantitative studies mostly focused on evaluating the impacts of interventions or examining associations between interventions and certain outcomes, qualitative studies described the intervention development or materials testing research and process evaluation. Mixed methods studies contributed to both describing the design and implementation of interventions and assessing the effectiveness of interventions.

The focus population of studies was mostly defined in a binary way: sex as males and females (Van Rossem and Meekers, 2000; Agha, 2002; Wilkins and Mak, 2007; Willoughby, 2013, 2015; Eastman-Mueller *et al*., 2019; Evans *et al*., 2019; Chou *et al*., 2020; Janssen *et al*., 2021), or men and women (Cho *et al*., 2004) and young women and their male partners (Friedman *et al*., 2014). One study (Garbers *et al*., 2016) defined males and females as gender identities whereas Habel *et al*. (2015) had female, male and transgender options in their questionnaire. Three studies focused only on females (Wagman, 1993; Meekers, 2000; Bull *et al*., 2008). The gender or sex of the target population was not mentioned in three studies (Zhang *et al*., 2017; Andrade *et al*., 2018; Aronowitz *et al.*, 2018).

Studies presented 14 interventions in total, of which 10 were named. The intervention ‘Horizon Jeunes’ was studied in two studies (Van Rossem and Meekers, 2000; Agha, 2002), ‘Adelante’ in two (Andrade *et al*., 2018; Evans *et al*., 2019), ‘Get Yourself Tested (GYT)’ in four (Friedman *et al*., 2014; Habel *et al*., 2015; Garbers *et al*., 2016; Eastman-Mueller *et al*., 2019) and ‘BrdsNBz’ in two (Willoughby, 2013, 2015). Additionally, one study (Agha, 2002) presented and summarized four interventions, of which one was ‘Horizon Jeunes’. Intervention duration varied from 1 month to 13 months, although the length was not reported in all articles, and it was unclearly described in some articles. The interventions were local, national or statewide, and the interventions were conducted in schools, communities and venues such as an outdoor music festival and a movie theatre.

**Fig. 2:**
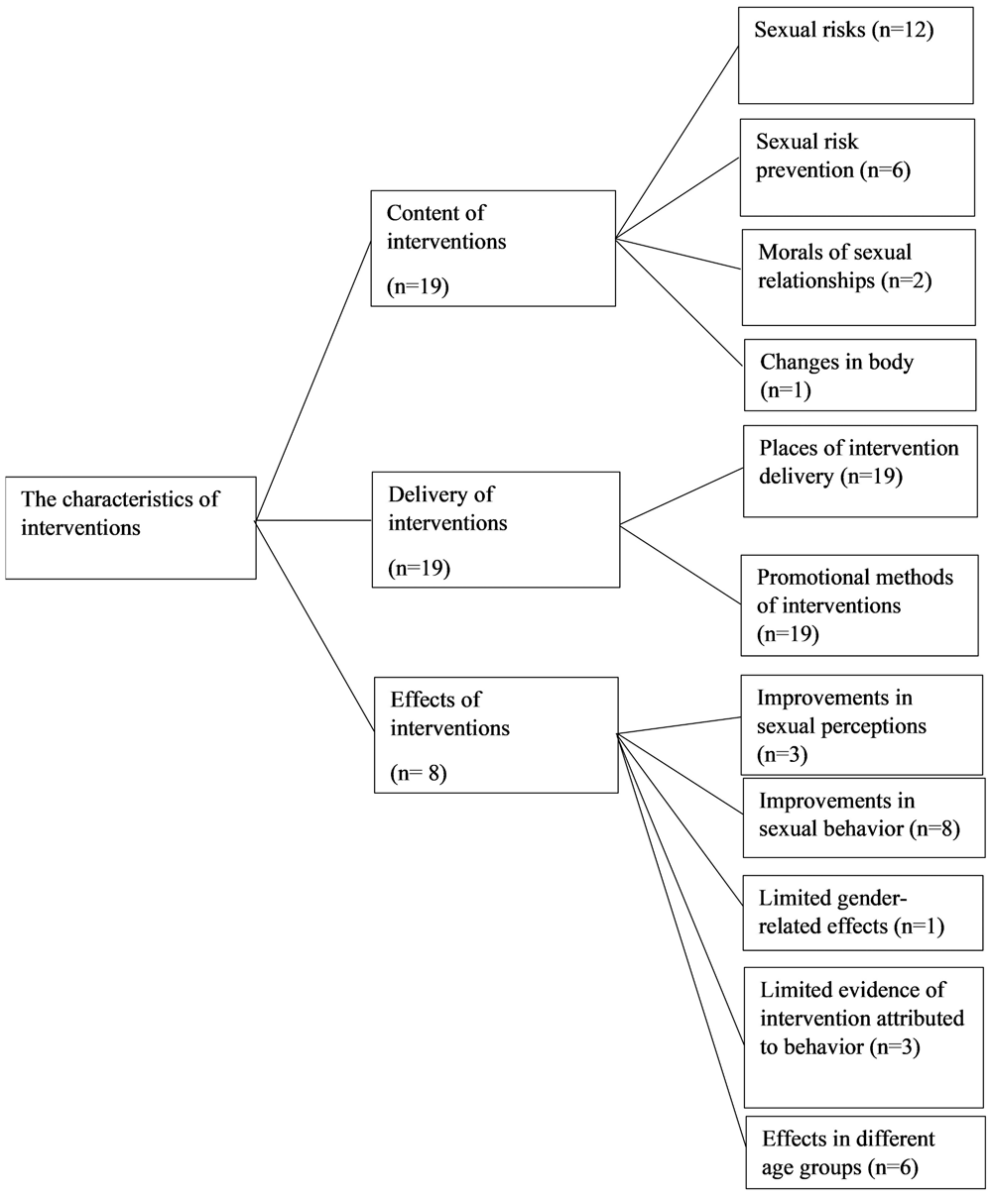
The categorization of results and the number of studies.

The content of interventions was addressed in four categories, delivery methods in two and the effects of interventions in five (Figure 2).

### Content of interventions

#### Sexual risks

Most studies (*n* = 12) focused on the risks (Wagman, 1993; Meekers, 2000; Van Rossem and Meekers, 2000; Agha, 2002; Wilkins and Mak, 2007; Friedman *et al*., 2014; Habel *et al*., 2015; Garbers *et al*., 2016; Zhang *et al*., 2017; Eastman-Mueller *et al*., 2019; Evans *et al*., 2019; Janssen *et al*., 2021). STIs and HIV were mentioned as risks, and unwanted pregnancies as an outcome of risky behaviour were often presented in the data. Additionally, sexual violence, sexual harassment and assault as topics regarding sexual risks emerged from the data (Agha, 2002; Andrade *et al*., 2018; Evans *et al*., 2019). Furthermore, peer pressure as a possible factor for sexual risk-taking was seen as one potential sexual risk matter (Meekers, 2000; Agha, 2002; Andrade *et al*., 2018). The risk approach was prevalent in studies conducted in North America, Australia and Africa.

#### Sexual risk prevention

The interventions also concentrated on sexual risk prevention. Many of the studies (*n* = 6) (Wilkins and Mak, 2007; Friedman *et al*., 2014; Habel *et al*., 2015; Garbers *et al*., 2016; Eastman-Mueller *et al*., 2019; Janssen *et al*., 2021) focused on promoting HIV and STI testing. Additionally, popularizing condom use was prevalent (Wagman, 1993; Meekers, 2000; Van Rossem and Meekers, 2000; Agha, 2002; Bull *et al*., 2008; Friedman *et al*., 2014) with one study marketing female and male condoms and targeting only women (Bull *et al*., 2008). Moreover, sexual communication emerged from the data and comprised topics such as how to tell your partner about STIs or pregnancy or how to have a dialogue about sexuality with one’s parents or healthcare providers. Males appeared to be passive in seeking sexual health information and relied on their female partners to share information about STIs or birth control (Cho *et al.*, 2004). Open communication about sexuality was encouraged as a preventive action or as an act for facing difficult situations (Agha, 2002; Cho *et al*., 2004).

#### Morals of sexual relationships

Intervention content also included morals of sexual relationship–related topics such as dating, romantic relationships and sex and love. In two studies (Van Rossem and Meekers, 2000; Agha, 2002), both from Africa, values such as the importance of being faithful to one’s partner, abstinence or fidelity were mentioned as desired principles. These values in relationships were seen as a preventive act for one’s sexual health and were provided in youth-oriented activities with peer educators at youth clubs and campaign messages announced on the radio.

#### Changes in body

One study from Asia mentioned body changes in puberty and valuing one’s body (Chou *et al*., 2020). This topic was taught at one of the formal education sessions provided by health education teachers and used role play as a teaching strategy. Body changes were observed from the reproductive changes and reproductive anatomy perspectives.

### Delivery methods of interventions

#### Places of intervention delivery

**Fig. 3:**
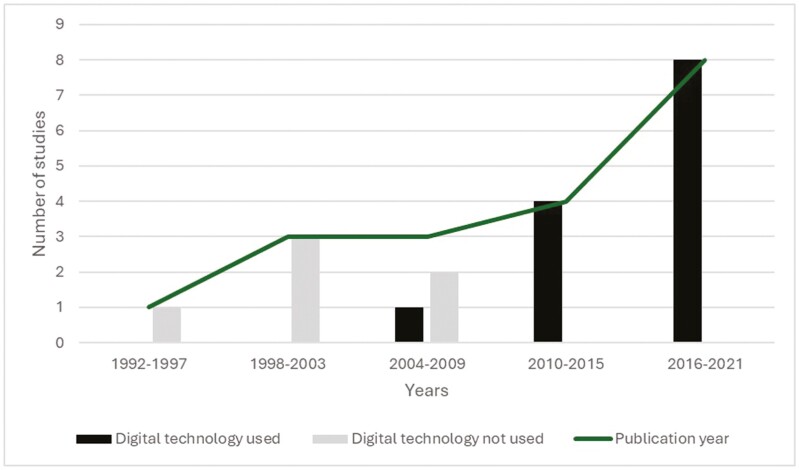
The use of digital technology in the included studies (*n* = 19).

Places of intervention delivery consisted of face-to-face delivery, intervention delivery through the Internet, intervention delivery through broadcast media, intervention delivery through print media and intervention delivery through out-of-home (OOH) media (BBA Mantra, 2017). Face-to-face delivery occurred at youth clubs in Africa (Van Rossem and Meekers, 2000; Agha, 2002), outdoor music festivals in Australia (Janssen *et al*., 2021), schools in North America and Asia (Friedman *et al*., 2014; Willoughby, 2015; Eastman-Mueller *et al*., 2019; Chou *et al*., 2020), campuses in North America (Habel *et al*., 2015; Aronowitz and Kim, 2018), and bars and nightclubs in North America (Wagman, 1993). Intervention delivery through social media and online was more common in recent studies (Figure 3). For example, blog posts, computer animations and websites were created (Wilkins and Mak, 2007; Andrade *et al*., 2018; Aronowitz and Kim, 2018; Eastman-Mueller *et al*., 2019; Chou *et al*., 2020). Traditional media (e.g. print media, television, SMS and radio) were used as one messaging channel in studies excluding two (Garbers *et al.*, 2016; Evans *et al.*, 2019). On the radio, for instance, call-in shows or weekly talk shows were held with guest experts (Meekers, 2000; Van Rossem and Meekers, 2000; Agha, 2002).

#### Promotional methods of intervention

To expose young people to interventions, various events and activities were mentioned in 11 interventions (Wagman, 1993; Meekers, 2000; Agha, 2002; Friedman *et al*., 2014; Habel *et al*., 2015; Garbers *et al*., 2016; Andrade *et al*., 2018; Aronowitz and Kim, 2018; Eastman-Mueller *et al*., 2019; Chou *et al*., 2020; Janssen *et al*., 2021). Special events, interactive occasions and educational activities with special promotional items formed the promotion of interventions. Special events comprised things such as Sex in the Dark Q&A event (Aronowitz and Kim, 2018), community meetings (Van Rossem and Meekers, 2000), lunchtime ‘Health Talks’ (Aronowitz and Kim, 2018), HIV testing events (Aronowitz and Kim, 2018) and sponsored events, such as concerts and soccer games (Agha, 2002). The special events offered young people opportunities to familiarize themselves with sexual health-related topics and services. Interactive occasions such as theatrical sketches (Van Rossem and Meekers, 2000), youth-generated videos (Andrade *et al*., 2018), outdoor music festival activations (Janssen *et al*., 2021), contests (Wagman, 1993; Andrade *et al*., 2018), quizzes (Eastman-Mueller *et al*., 2019; Chou *et al*., 2020), wheel of sex game (Aronowitz and Kim, 2018), poster competitions (Chou *et al*., 2020), roleplays (Chou *et al*., 2020), activations facilitated by trained peers (Janssen *et al*., 2021), gaming (Zhang *et al*., 2017), debates (Chou *et al*., 2020) and discussion groups (Agha, 2002) aimed at involving young people themselves to be actors and vigorous participants in the scene. Educational activities consisted of educational theatre (Agha, 2002), film presentation about the topic (Agha, 2002), videos (Andrade *et al*., 2018; Eastman-Mueller *et al*., 2019), condom use demonstration (Van Rossem and Meekers, 2000; Agha, 2002) and peer educations (Meekers, 2000; Van Rossem and Meekers, 2000; Agha, 2002), and they had a strong educative basis. Special promotional items such as posters, flyers, condoms, takeaway information cards, pens, pins and gift incentives were distributed at events (Willoughby, 2013). The delivery methods appeared to be versatile, innovative and multi-channelled.

### Effects of interventions

The effects of interventions on young people’s sexual health were assessed in eight studies (Agha, 2002; Cho *et al*., 2004; Wilkins and Mak, 2007; Bull *et al*., 2008; Willoughby, 2015; Evans *et al*., 2019; Chou *et al*., 2020; Janssen *et al*., 2021). Additionally, the effects of the Get Yourself Tested (GYT) intervention were evaluated in four studies: one focused on the high school–college comparison perspective (Eastman-Mueller *et al*., 2019), one focused on the campaign program level (Friedman *et al*., 2014), one evaluated the intervention from the Black and Latino sexual minority youth perspective (Garbers *et al*., 2016) and one study performed a pilot evaluation (Habel *et al*., 2015). To determine the effectiveness of interventions, various statistical analyses such as the paired samples *t*-test to measure the effectiveness of the intervention for the promotion of adolescents’ sexual knowledge and attitudes (Chou, 2020), permutation test to measure awareness, attitudes and use of male and female condoms (Bull *et al.*, 2008) and multivariate logistic regression for measuring both perceptions and behaviours related to sexual health were utilized (Agha, 2002). The effects of interventions were discovered in improvements in sexual perceptions and sexual behaviour. However, limited gender-related effects relating to biological sex and limited evidence of intervention attributed to behaviour also emerged from the data.

#### Improvements in sexual perceptions

Improvements in sexual perceptions were found in increased sexual knowledge, improved sexual attitudes and sexual awareness (Chou *et al*., 2020). Knowledge of birth control methods increased (Van Rossem and Meekers, 2000), and lower risky attitudes towards sex among females (Evans *et al*., 2019) were reported. Increased awareness of sexual risks, such as the risk of pregnancy or HIV prevention, was mentioned in the data (Van Rossem and Meekers, 2000).

#### Improvements in sexual behaviour

Improvements in sexual behaviour were seen in improved contraceptive use (Agha, 2002) and increased sexual discussion (Meekers, 2000; Van Rossem and Meekers, 2000). Those interventions that concentrated solely on STI testing promotion were effective in increasing testing participation. Five STI testing interventions (Wilkins and Mak, 2007; Friedman *et al*., 2014; Garbers *et al*., 2016; Eastman-Mueller *et al*., 2019; Janssen *et al*., 2021) out of six (Wilkins and Mak, 2007; Friedman *et al*., 2014; Habel *et al*., 2015; Garbers *et al*., 2016; Eastman-Mueller *et al*., 2019; Janssen *et al*., 2021) contributed to enhanced STI screening. Also, one study (Aronowitz and Kim, 2018) that was not concentrating only on testing but was a multi-level intervention reported HIV testing to be tripled. Other improvements in behaviour were related to contraceptive and condom use (Meekers, 2000; Agha, 2002). Positive impacts on oral contraceptive use and an increased proportion of female youth using condoms were reported. Sexual discussion increased as an effect of interventions (Van Rossem and Meekers, 2000). The reported discussion topics regarded contraception and other sexual issues. Also, awareness of an STI test promotion intervention was associated with talk about sexual health and STIs between relationship partners for college students (Eastman-Mueller *et al*., 2019). The effect on behaviour was also seen in one study (Agha, 2002) where men were less likely after the intervention than before to have multiple or casual partners. Interventions that led to these results were conducted in Africa, lasted 8–13 months, and were multi-channelled: they included peer education, youth clubs with discussion groups and condom use demonstrations, mass media advertising and youth-friendly outlets with educated retailers providing sexual health counselling.

#### Limited gender-related effects

Some effects of interventions on perceptions and behaviour were observed by gender. Improvements in sexual perceptions regarding the benefits and barriers to preventive behaviour were seen in women along with improved contraceptive use. These effects were more limited among men (Agha, 2002). Other genders were not distinguished in the evaluation of the effects.

#### Limited evidence of intervention attributed to behaviour

Some interventions reported neutral effects (Bull *et al*., 2008). According to the data, some desired behaviour changes might have happened, but it was not always evident that the change was attributed to the intervention. This observation regarded condom use (Bull *et al*., 2008). A quasi-experimental study (Meekers, 2000) did not find significant changes in either intervention or comparison groups. One college that was hosting a promotional testing event did not see a higher proportion of tested students (Habel *et al*., 2015).

#### Effects in different age groups

Effects varied across different age groups. Among young participants aged 13–14 years, sexual perceptions were more often improved (Chou, 2020). In older participants, with an average age of 17 years, sexual behaviour regarding contraceptive use increased (Agha, 2002). Among young people in their twenties, neutral effects or improved behaviour regarding sexual discussion and STI screening were observed (Cho *et al*., 2004; Wilkins and Mak, 2007; Bull *et al*., 2008; Janssen *et al*., 2021).

## DISCUSSION

The objective of this scoping review was to identify and map the existing circumstances on how social marketing has been used in sexual health promotion targeting young people. Specifically, the content, delivery methods and effects of interventions on sexual health were researched.

The included studies utilized a wide range of social marketing strategies. However, of the intervention types, the educational form was emphasized. The key finding was that although the implementation techniques of social marketing interventions were versatile, innovative and multi-channelled, the content of interventions often followed rather traditional subject matters. The approach was mostly preventive, concentrating on the risks instead of seeing sexuality as a positive resource or a part of being human. Emphasizing physical health outcomes and risks is common in studies researching adolescent sexual and reproductive health and rights (Liang *et al*., 2019), although newly published literature has now raised themes such as pleasure into discussion (Beckmeyer *et al.*, 2021).

As sexual risks and their prevention dominated the content of this study, other essential sex education themes were less prevalent. Reflecting the definition of sexual health with its’ physical, emotional, mental and social well-being about sexuality (World Health Organization, 2021), many themes that fall into the definition, such as equal romantic and sexual relationships, pleasure, mental and social well-being and self-esteem were not seen in the data. Sexual rights were mentioned in only one study (Chou *et al*., 2020), consent in one (Aronowitz and Kim, 2018), sexual diversity in one (Chou *et al*., 2020), gender equality in one (Chou *et al*., 2020) and gender identity in one (Garbers *et al*., 2016). The previously mentioned studies have been published more recently, and this may signal the broadening of perspectives. At the same time, the chosen content reflects values that are appreciated in a society at certain times. The surrounding culture affects how sexuality is seen and what content is emphasized (Agocha *et al*., 2014). For instance, all the studies of this review from Africa were conducted 20 years ago. High HIV prevalence, gender inequalities and male domination in sex adequately explain the chosen preventive content. The culture sensitivities must be observed when interpreting the results.

Based on the results, it seems that with a younger target population, the effects of interventions are seen in sexual perceptions, whereas with older ones the effects are neutral or relate to sexual behaviour. The findings regarding sexual behaviour are consistent with a previous study (Friedman *et al*., 2016). Even if this study found positive effects on young people’s sexual perceptions and behaviour after exposure to the interventions, limited evidence of intervention effectiveness and limited gender-related effects were also reported. The latter follows Wakhisi *et al*. (2011) who announced the impact on male participants’ sexual behaviour was minimal in their review.

To obtain a wide understanding of the current state, this review included a heterogeneous set of publications with varying study methodologies, settings and publication types. This is in line with the aim of scoping reviews (Arksey and O’Malley, 2005).

Relatively few publications of social marketing promoting young people’s sexual health have been executed. Regarding the main global challenges in young people’s sexual health, such as inequalities in key indicators, increase in intimate partner violence, reproductive cancer and STIs, a gap in the literature is recognized and additional social marketing research is needed.

According to the World Bank, over 40% of the global population is under the age of 25 (Pirlea *et al*., 2023). Adolescents’ world is changing rapidly towards becoming more urban and mobile (Liang *et al*., 2019). In a fast-paced digital and global environment, the sexual health literacy demands are vast. Hence, sexual health promotion must keep up with young people’s rapidly changing needs, and this requires continually updating the content and delivery methods of interventions. This study is important because the body of knowledge on the content, delivery methods and effects of social marketing interventions targeting young people is limited and only emphasizes risks and risk prevention.

### Limitations

There are some limitations to consider when interpreting the results. First, the included studies of the scoping review were unevenly distributed as most were implemented in North America. This may have caused some lingual and areal bias. According to this study, the focus has been minimal in Africa, Australia and Asia, whereas South America and Europe were not represented at all. Therefore, the findings are not generalizable universally. Second, several studies included in this review focused on the same interventions. Third, the age distribution in this study was rather large. Young people may have different kinds of needs than young adults and these possible differences are not distinguished here. Fourth, it is possible that some related but less common search terms have remained unused and the chosen inclusion criteria of empirical studies have limited the results and thus relevant papers may have remained unfound. Finally, in this review, the studies self-identified themselves as social marketing programs. Because of that, the use of social marketing principles may have varied in the included studies. It is evident from previous studies that behavioural change objectives, consumer research and the marketing mix theory play a significant role in increasing program effectiveness (Carins and Rundle-Thiele, 2014; Kubacki *et al*., 2017).

## CONCLUSION

This study increases understanding of and provides insight into the topic that has received very little research by identifying and mapping the existing circumstances on how social marketing has been used in sexual health promotion targeting young people. The use of social marketing offers a valid approach that outlines innovative and versatile ways to promote young people’s sexual health. Digitalization and the use of technology have become more widespread over time and provide platforms for social marketing to influence behaviours. When young people are in focus their use is apparent as sexual health promotion must occur where the young people are. The results of this study prove that the content of social marketing interventions does not always respond to the current societal needs. Future research is needed to investigate what guides the choice of content and delivery methods in social marketing interventions and what role sexual health perspectives and needs of young people serve in the process. Additionally, the effectiveness of sexual health social marketing interventions targeting young people requires more profound evidence. The findings of this review are beneficial in raising awareness of the phenomena by summarizing the existing knowledge and in planning both sexual health-related social marketing interventions and sexual health campaigns and programs of other types.

## Supplementary Material

daae106_suppl_Supplementary_Material

## References

[CIT0001] Agha, S. (2002) A quasi-experimental study to assess the impact of four adolescent sexual health interventions in sub-Saharan Africa. International Family Planning Perspectives, 28, 67–70.

[CIT0002] Agocha, V. B., Asencio, M. and Decena, C. U. (2014) Sexuality and culture. In Tolman, D. L., Diamond, L. M. and Bauermeister, J. A. et al. (eds), APA Handbook of Sexuality and Psychology, Vol. 2: Contextual Approaches. American Psychological Association, Washington, pp. 183–228. http://content.apa.org/books/14194-006 (last accessed 17 February 2023).

[CIT0003] Andrade, E. L., Evans, W. D., Barrett, N. D., Cleary, S., Edberg, M., Alvayero, R. et al. (2018) Development of the place-based Adelante social marketing campaign for prevention of substance use, sexual risk and violence among Latino immigrant youth. Health Education Research, 33, 125–144. 29329436 10.1093/her/cyx076 PMC6658711

[CIT0005] Arksey, H. and O’Malley, L. (2005) Scoping studies: towards a methodological framework. International Journal of Social Research Methodology, 8, 19–32.

[CIT0006] Aronowitz, T., Kim, B., Vu, P. and Bergeron, A. (2018) Engaging college students in a substance misuse & sexual health intervention using social marketing principles. Applied Nursing Research, 44, 88–92. 30389066 10.1016/j.apnr.2018.10.006

[CIT0007] BBA Mantra. (2017) Media—types of media, print, broadcast, outdoor, internet. https://bbamantra.com/media-types-characteristics/ (last accessed 17 January 2023).

[CIT0063] Beckmeyer, J. J., Herbenick, D., Fu, T. C., Dodge, B., Fortenberry, D. J. (2021) Pleasure during adolescents’ most recent partnered sexual experience: findings from a U.S. probability survey. *Archive of Sexual Behavior*, 50, 2423–2434. 10.1007/s10508-021-02026-4 34373980

[CIT0008] Bull, S. S., Posner, S. F., Ortiz, C., Beaty, B., Benton, K., Lin, L. et al. (2008) POWER for reproductive health: results from a social marketing campaign promoting female and male condoms. Journal of Adolescent Health, 43, 71–78. 10.1016/j.jadohealth.2007.12.009 18565440

[CIT0009] Carins, J. E. and Rundle-Thiele, S. R. (2014) Eating for the better: a social marketing review (2000–2012). Public Health Nutrition, 17, 1628–1639. 23711161 10.1017/S1368980013001365 PMC10282391

[CIT0010] Cho, H., Oehlkers, P., Mandelbaum, J., Erlund, K. and Zurek, M. (2004) The Healthy Talk family planning campaign of Massachusetts: a communication-centered approach. Health Education, 104, 314–325.

[CIT0011] Chou, L. -N., Shen, I. C., Chu, T. -P. and Chen, M. (2020) Effectiveness of a school-based social marketing intervention to promote adolescent sexual health. Health Education Journal, 79, 34–45.

[CIT0012] Coll, L., O’Sullivan, M. and Enright, E. (2018) ‘The Trouble with Normal’: (re)imagining sexuality education with young people. Sex Education, 18, 157–171.

[CIT0013] Council of Europe. (2020) Comprehensive sexuality education protects children and helps build a safer, inclusive society. The commissioner’s human rights comments. www.coe.int/en/web/commissioner/-/comprehensive-sexuality-education-protects-children-and-helps-build-a-safer-inclusive-society (last accessed 18 January 2023).

[CIT0014] Eastman-Mueller, H., Habel, M., Oswalt, S. and Liddon, N. (2019) Get yourself tested (GYT) campaign: investigating campaign awareness and behaviors among high school and college students. Health Education and Behavior, 46, 63–71. 30064270 10.1177/1090198118788617 PMC6736513

[CIT0015] Engel, E. (2023) Young peoples’ perceived benefits and barriers of sexual health promotion on social media—a literature review. International Journal of Health Promotion and Education, 1, 1–20.

[CIT0016] Evans, W., Andrade, E., Barrett, N., Snider, J., Cleary, S. and Edberg, M. (2019) Outcomes of the Adelante community social marketing campaign for Latino youth. Health Education Research, 34, 471–482. 31106344 10.1093/her/cyz016 PMC7962720

[CIT0017] Forsyth, S. and Rogstad, K. (2015) Sexual health issues in adolescents and young adults. Clinical Medicine (London, England), 15, 447–451. 26430183 10.7861/clinmedicine.15-5-447 PMC4953229

[CIT0018] Friedman, A., Bozniak, A., Ford, J., Hill, A., Olson, K., Ledsky, R. et al. (2014) Reaching youth with sexually transmitted disease testing: building on successes, challenges, and lessons learned from local Get yourself tested campaigns. Social Marketing Quarterly, 20, 116–138. 31749662 10.1177/1524500414530386 PMC6866650

[CIT0019] Friedman, A., Kachur, R., Noar, S. and McFarlane, M. (2016) Health communication and social marketing campaigns for sexually transmitted disease prevention and control: what is the evidence of their effectiveness? Sexually Transmitted Diseases, 43, S83–S101. 26779691 10.1097/OLQ.0000000000000286

[CIT0020] Garbers, S., Friedman, A., Martinez, O., Scheinmann, R., Bermudez, D., Silva, M. et al. (2016) Adapting the get yourself tested campaign to reach Black and Latino sexual-minority youth. Health Promotion Practice, 17, 739–750. 27225216 10.1177/1524839916647329 PMC4980262

[CIT0021] Habel, M. A., Haderxhanaj, L., Hogben, M., Eastman-Mueller, H., Chesson, H. and Robers, C. (2015) Does your college campus GYT? Evaluating the effect of a social marketing campaign designed to raise STI awareness and encourage testing. Cases in Public Health Communication and Marketing, 8, 51–70. 31749899 PMC6866652

[CIT0022] Harris, A. L. (2011) Media and technology in adolescent sexual education and safety. Journal of Obstetric, Gynecologic, & Neonatal Nursing, 40, 235–242. 10.1111/j.1552-6909.2011.01218.x 21284726

[CIT0023] Janssen, M., Okeke, S., Murray, C., Ewing, M., Lu, H., Bourne, C. et al. (2021) STI testing among young people attending music festivals in New South Wales, Australia: exploring the client segmentation concept in the ‘Down to Test’ program. Sexual Health, 18, 405–412. 34782058 10.1071/SH21101

[CIT0024] The Joanna Briggs Institute. (2023) Methodology for JBI scoping reviews. In Aromataris, E. (ed), The JoannaBriggs Institute Reviewers’ Manual 2015. The Joanna Briggs Institute, Australia.

[CIT0025] Kar, S., Choudhury, A. and Singh, A. (2015) Understanding normal development of adolescent sexuality: a bumpy ride. Journal of Human Reproductive Sciences, 8, 70. 26157296 10.4103/0974-1208.158594 PMC4477452

[CIT0026] Kotler, P. and Zaltman, G. (1971) Social marketing: an approach to planned social change. Journal of Marketing, 35, 3–12. 12276120

[CIT0027] Kubacki, K., Ronto, R., Lahtinen, V., Pang, B. and Rundle-Thiele, S. (2017) Social marketing interventions aiming to increase physical activity among adults: a systematic review. Health Education, 117, 69–89.

[CIT0028] Kyngäs, H., Mikkonen, K. and Kääriäinen, M. (2019) The Application of Content Analysis in Nursing Science Research. Springer International Publishing, Cham.

[CIT0029] Lee, N., Kotler, P. and Colehour J. (2023) Social Marketing BehaviorChange for Good, 7th edition. Sage Publications, California.

[CIT0030] Liang, M., Simelane, S. and Fillo, G. (2019) The state of adolescent sexual and reproductive health. Journal of Adolescent Health, 2019, 3–15. 10.1016/j.jadohealth.2019.09.015 31761002

[CIT0031] Loaiza, E. and Liang, M. (2013) Adolescent pregnancy: a review of the evidence. www.unfpa.org/publications/adolescent-pregnancy (last accessed 6 June 2022).

[CIT0032] Martin, P., Cousin, L., Gottot, S., Bourmaud, A., de La Rochebrochard, E. and Alberti, C. (2020) Participatory interventions for sexual health promotion for adolescents and young adults on the internet: systematic review. Journal of Medical Internet Research, 22, e15378. 32735217 10.2196/15378 PMC7428916

[CIT0033] Meekers, D. (2000) The effectiveness of targeted social marketing to promote adolescent reproductive health: the case of Soweto, South Africa. Journal of HIV/AIDS Prevention & Education for Adolescents & Children, 3, 73–92. 11063059

[CIT0034] Mehta, S. D. and Seeley, J. (2020) Grand challenges in adolescent sexual and reproductive health. Frontiers in Reproductive Health, 2, 2. 36304711 10.3389/frph.2020.00002 PMC9580643

[CIT0035] Merrick, J., Tenenbaum, A. and Omar, H. A. (2013) Human sexuality and adolescence. Frontiers in Public Health, 1, 41. 24350210 10.3389/fpubh.2013.00041 PMC3859969

[CIT0036] Messer, L. C., Shoe, E., Canady, M., Sheppard, B. K. and Vincus, A. (2011) Reported adolescent sexual norms and the development of a social marketing campaign to correct youth misperceptions. Journal of Children and Poverty, 17, 45–63.

[CIT0037] Munn, Z., Peters, M. D. J., Stern, C., Tufanaru, C., McArthur, A. and Aromataris, E. (2018) Systematic review or scoping review? Guidance for authors when choosing between a systematic or scoping review approach. BMC Medical Research Methodology, 18, 143. 30453902 10.1186/s12874-018-0611-x PMC6245623

[CIT0038] National Social Marketing Centre. (2023) What is social marketing? The NSMC. www.thensmc.com/what-social-marketing (last accessed 16 January 2023).

[CIT0039] Pakarinen, M., Kylmä, J., Helminen, M. and Suominen, T. (2020) Attitudes, knowledge and sexual behavior among Finnish adolescents before and after an intervention. Health Promotion International, 35, 821–830. 31436843 10.1093/heapro/daz074

[CIT0040] Pirlea, A., Serajuddin, D., Wadhwa, M. and Welch, E. (2023) Atlas of sustainable development goals 2023. World Bank. License: Creative commons attribution, Washington, DC. https://datatopics.worldbank.org/sdgatlas/ (last accessed 16 January 2023).

[CIT0041] Ponsford, R., Bragg, S., Meiksin, R., Tilouche, N., Van Dyck, L., Sturgess, J. et al. (2022) Feasibility and acceptability of a whole-school social-marketing intervention to prevent unintended teenage pregnancies and promote sexual health: evidence for progression from a pilot to a phase III randomised trial in English secondary schools. Pilot and Feasibility Studies, 8, 52. 35246272 10.1186/s40814-022-00971-y PMC8895534

[CIT0042] Proquest. (2024) What is RefWorks. https://proquest.libguides.com/refworks#s-lg-box-26056729 (last accessed 12 April 2024).

[CIT0043] Purdy, C. (2011) Using the Internet and social media to promote condom use in Turkey. Reproductive Health Matters, 19, 157–165. 21555096 10.1016/S0968-8080(11)37549-0

[CIT0044] Riddell, J., Cleary, A., Dean, J. A., Flowers, P., Heard, E., Inch, Z. et al. (2024) Social marketing and mass media interventions to increase sexually transmissible infections (STIs) testing among young people: social marketing and visual design component analysis. BMC Public Health, 24, 620. 38408945 10.1186/s12889-024-18095-8 PMC10898181

[CIT0045] Sanz-Lorente, M., Wanden-Berghe, C., Castejón-Bolea, R. and Sanz-Valero, J. (2018) Web 2.0 tools in the prevention of curable sexually transmitted diseases: scoping review. Journal of Medical Internet Research, 20, e113. 29567633 10.2196/jmir.8871 PMC5887040

[CIT0046] Shell-Duncan, B. and Naik, R. (2016) A State-of-the-Art Synthesis on Female Genital Mutilation/Cutting What Do We Know Now? Population Council, New York.

[CIT0062] Slater, C., Robinson, A. J. (2014) Sexual health in adolescents. *Clinics in Dermatology*, 32, 189–195. 24559553 10.1016/j.clindermatol.2013.08.002

[CIT0047] Sweat, M., Denison, J., Kennedy, C., Terdow, V. and O’Reilly, K. (2012) Effects of condom social marketing on condom use in developing countries: a systematic review and meta-analysis, 1990–2010. Bulletin of the World Health Organization, 90, 613–622. 22893745 10.2471/BLT.11.094268 PMC3417793

[CIT0048] Tricco, A. C., Lillie, E., Zarin, W., O’Brien, K. K., Colquhoun, H., Levac, D. et al. (2018) PRISMA extension for scoping reviews (PRISMA-ScR): checklist and explanation. Annals of Internal Medicine, 169, 467–473. 30178033 10.7326/M18-0850

[CIT0049] United Nations Children’s Fund. (2018) Child Marriage Latest Trends and Future Prospects. UNICEF, New York. https://data.unicef.org/resources/child-marriage-latest-trends-and-future-prospects/

[CIT0050] Van Rossem, R. and Meekers, D. (2000) An evaluation of the effectiveness of targeted social marketing to promote adolescent and young adult reproductive health in Cameroon. AIDS Education and Prevention, 12, 383–404. 11063059

[CIT0051] Wagman, L. M. (1993) A health department’s response to AIDS. Condomania: a public education intervention. Canadian Journal of Public Health, 84, 62–65. 8481875

[CIT0052] Wakhisi, A. S., Allotey, P., Dhillon, N. and Reidpath, D. (2011) The effectiveness of social Marketing in reduction of teenage pregnancies: a review of studies in developed countries. Social Marketing Quarterly, 1, 56–90.

[CIT0053] Weinreich, N. (2011) Hands-On Social Marketing: A Step-by-Step Guide to Designing Change for Good. Sage Publications, California.

[CIT0054] Wildsmith, E. and Vaughn, B. (2013) Dating and sexual relationships. US Department of Health and Human Services. https://opa.hhs.gov/adolescent-health/healthy-relationships-adolescence (last accessed 20 January 2023).

[CIT0055] Wilkins, A. and Mak, D. B. (2007) … sending out an SMS: an impact and outcome evaluation of the Western Australian Department of Health’s 2005 chlamydia campaign. Health Promotion Journal of Australia, 18, 113–120. 17663646 10.1071/he07113

[CIT0056] Wilkinson, A., Pedrana, A., El-Hayek, C., Vella, A., Asselin, J., Batrouney, C. et al. (2016) The impact of a social marketing campaign on HIV and sexually transmissible infection testing among men who have sex with men in Australia. Sexually Transmitted Diseases, 43, 49–56. 26650997 10.1097/OLQ.0000000000000380

[CIT0057] Willoughby, J. F. (2013) Everyone has questions: developing a social marketing campaign promoting a sexual health text message service. Social Marketing Quarterly, 19, 265–278.

[CIT0058] Willoughby, J. F. (2015) Effectiveness of a social marketing campaign promoting use of a sexual health text service by teens. Journal of Health Communication, 20, 1206–1213. 26010464 10.1080/10810730.2015.1018586

[CIT0059] World Health Organization. (2016) Sexual and reproductive health literacy and the SDGs. www.who.int/healthpromotion/conferences/9gchp/sexual-reproductive-health-literacy/en/ (last accessed 27 June 2024).

[CIT0060] World Health Organization. (2021) Sexuality education. Sexuality and reproductive health 2021. www.who.int/health-topics/sexual-health#tab=tab_1 (last accessed 24 January 2023).

[CIT0061] Zhang, Q., Huhn, K. J., Tan, A., Douglas, R., Li, H., Murti, M. et al. (2017) ‘Testing is healthy’ TimePlay campaign: evaluation of sexual health promotion gamification intervention targeting young adults. Canadian Journal of Public Health, 108, 85–90. 10.17269/CJPH.108.5634 PMC6972345 28425904

