# Supplementary material for: Sexual health interventions with social marketing approach targeting young people: a scoping review
Source: Health Promot Int. 2024 Aug 16;39(4):daae106. doi: 10.1093/heapro/daae106 (PMC11327497; doi:10.1093/heapro/daae106)
Supplement: daae106_suppl_Supplementary_Material [file daae106_suppl_supplementary_material.docx]

SUPPLEMENTAL FILE 1

SUPPLEMENTAL TABLE FOR SEARCH STRATEGIES

| **DATABASE** | **SEARCH TERMS** |
| --- | --- |
| PubMed | ("social market*" OR "Social Marketing"[Mesh])  AND ("sexual health*" OR "sex educati*" OR "sexual educati*" OR "sexual well-being") |
| Eric | "social market*" AND "sexual health*" OR “sex educati*” OR “sexual educati*” OR "sexual well-being" |
| Web of Science | ((ALL=("social market*")) AND ALL=("sexual health*")) OR ALL=("sexual educati*") |
| Scopus | ( TITLE-ABS-KEY ( "social marketing" ) AND TITLE-ABS-KEY ( "sexual health*" OR "sex educati*" OR "sexual educati*" OR "sexual well-being" ) ) |
| Cinahl | "social market*" AND "sexual health*" OR “sex educati*” OR “sexual educati*” OR "sexual well-being" |
| PsycInfo | "social market*" AND "sexual health*" OR “sex educati*” OR “sexual educati*” OR "sexual well-being" |
